# A High-Protein Diet Reduces Weight Gain, Decreases Food Intake, Decreases Liver Fat Deposition, and Improves Markers of Muscle Metabolism in Obese Zucker Rats

**DOI:** 10.3390/nu9060587

**Published:** 2017-06-08

**Authors:** William W. French, Sami Dridi, Stephanie A. Shouse, Hexirui Wu, Aubree Hawley, Sun-Ok Lee, Xuan Gu, Jamie I. Baum

**Affiliations:** 1 Department of Biological Sciences, University of Arkansas, Fayetteville, AR 72704, USA; wwfrench@email.uark.edu; 2 Center for Poultry Excellence, Division of Agriculture, University of Arkansas, Fayetteville, AR 72704, USA; dridi@uark.edu; 3 Department of Food Science, Institute of Food Science and Engineering, Center for Human Nutrition, Division of Agriculture, University of Arkansas, Fayetteville, AR 72704, USA; sashouse@uark.edu (S.A.S.); hw014@email.uark.edu (H.W.); alworden@email.uark.edu (A.H.); sunok@uark.edu (S.-O.L.); xuangu@uark.edu (X.G.)

**Keywords:** protein, diabetes, obesity, muscle, liver, body composition, diet

## Abstract

A primary factor in controlling and preventing obesity is through dietary manipulation. Diets higher in protein have been shown to improve body composition and metabolic health during weight loss. The objective of this study was to examine the effects of a high-protein diet versus a moderate-protein diet on muscle, liver and fat metabolism and glucose regulation using the obese Zucker rat. Twelve-week old, male, Zucker (fa/fa) and lean control (Fa/fa) rats were randomly assigned to either a high-protein (40% energy) or moderate-protein (20% energy) diet for 12 weeks, with a total of four groups: lean 20% protein (L20; *n* = 8), lean 40% protein (L40; *n* = 10), obese 20% protein (O20; *n* = 8), and obese 40% protein (O40; *n* = 10). At the end of 12 weeks, animals were fasted and euthanized. There was no difference in food intake between L20 and L40. O40 rats gained less weight and had lower food intake (*p* < 0.05) compared to O20. O40 rats had lower liver weight (*p* < 0.05) compared to O20. However, O40 rats had higher orexin (*p* < 0.05) levels compared to L20, L40 and O20. Rats in the L40 and O40 groups had less liver and muscle lipid deposition compared to L20 and L40 diet rats, respectively. O40 had decreased skeletal muscle mechanistic target of rapamycin complex 1 (mTORC1) phosphorylation and peroxisome proliferator-activated receptor gamma (PPARγ) mRNA expression compared to O20 (*p* < 0.05), with no difference in 5′ AMP-activated protein kinase (AMPK), eukaryotic translation initiation factor 4E binding protein 1 (4EBP1), protein kinase B (Akt) or p70 ribosomal S6 kinase (p70S6K) phosphorylation. The data suggest that high-protein diets have the potential to reduce weight gain and alter metabolism, possibly through regulation of an mTORC1-dependent pathway in skeletal muscle.

## 1. Introduction

Obesity is a major public health concern [1] and is associated with development of metabolic diseases such as cardiovascular disease, non-alcoholic fatty liver disease, and type 2 diabetes mellitus (T2DM) [2]. A primary factor in controlling and preventing obesity and associated chronic diseases is through dietary manipulation. One such dietary approach is the consumption of a diet higher in protein. The recommended acceptable macronutrient distribution range (AMDR) for carbohydrate intake is 45–65% of energy intake, and for protein, 10–35% of energy intake [3]. Diets high in protein (>30% of energy intake) have been shown to promote weight loss, improve body composition, and regulate glycemic response in both human [4,5,6,7,8] and animal models [9,10]. Despite the positive effects diets high in protein have on weight loss and weight maintenance, long-term observational studies report an association between high protein intake and a higher risk of developing T2DM [11,12,13], specifically diets with high total protein intake [13,14] and high animal protein intake [13,14,15]. This is important since muscle plays a central role in whole body protein metabolism and disrupted muscle metabolism is associated with the development of many common chronic diseases such as T2DM and cardiovascular disease [16].

The primary role of protein in the diet is to provide amino acids required for synthesis of new proteins, especially the nine essential amino acids, which includes the branched-chain amino acids (BCAAs) leucine, valine and isoleucine [17,18]. However, BCAAs play a metabolic role beyond the requirements for protein synthesis [19,20]. BCAAs are not only substrates for various metabolic pathways, such lipid and glucose metabolism [21,22,23], but can also serve as signaling molecules controlling signal transduction pathways and gene transcription [21,24,25,26]. In many cases the activation of metabolic pathways is in proportion to dietary intake of amino acids [19], for example, tryptophan, which is a precursor for the appetite-modulating neurotransmitter serotonin [27,28] or leucine, which modulates glycemic control, possibly through phosphorylation of phosphatidylinositol-3 kinase [24,29,30] and regulates muscle protein synthesis via mechanistic target of rapamycin complex 1 (mTORC1) [31,32].

mTORC1 is a positive regulator of protein and lipid synthesis, and controls cell metabolism, size and proliferation depending on the availability of nutrients, growth factors and cellular energy status [33,34,35]. Data suggest that mTORC1 also mediates adipogenesis via peroxisome proliferator-activated receptor-gamma (PPARγ) [36,37], a master regulator of adipogenesis and lipid metabolism [37]. mTORC1 has also been associated with PPARγ-induced stimulation of adipose tissue lipid uptake and fat accretion [37]. In addition, there is some evidence that PPARγ activity may dependent on amino acid status [36], similar to mTORC1. These findings suggest that the health benefits observed in individuals following a higher protein diet may be mediated by both mTORC1 and PPARγ.

Although the health benefits of diets higher in protein are well-established, the mechanisms of action responsible for the changes in body weight, body composition and glycemic control are not well-characterized. Therefore, the objective of this study was to examine the effects of a long-term, high-protein diet (40% protein; slightly above the AMDR) versus a moderate-protein diet (20% protein; within the AMDR) on muscle, liver and adipose tissue metabolism, body composition, and glucose regulation using the Zucker rat as a model for obesity and T2DM.

## 2. Materials and Methods

### 2.1. Experimental Design

The Animal Care and Use Committee (IACUC) at the University of Arkansas approved the experimental protocol. Twelve-week old, male, Zucker (fa/fa; O, obese) and lean control (Fa/fa; L, lean) rats were used in this study (Envigo, Indianapolis, IN, USA). Animals were housed two per cage in a sedentary environment with a 12-h light/dark cycle. Each animal cage was randomly assigned to one of two experimental diets (Supplemental Table S1; purchased premade from Envigo, Indianapolis, IN, USA), moderate protein (20% protein; catalog #TD. 90018) or high protein (40% protein; catalog #TD. 91352), for 12 weeks, with a total of four groups: lean 20% protein (L20; *n* = 8), lean 40% protein (L40; *n* = 10), obese 20% protein (O20; *n* = 8), and obese 40% protein (O40; *n* = 10). Animals were provided ad libitum access to their specific diet and water throughout the duration of the study. Body weights were measured daily, and food intake was measured four times per week. After 12 weeks, animals were fasted for 8 h and euthanized using an injection of sodium pentobarbital (80 mg/kg body weight) followed by decapitation. Trunk blood was collected and centrifuged at 1800× *g* for 10 min at 4 °C. Plasma was collected and immediately frozen in liquid nitrogen and stored at −80 °C until analysis. Gastrocnemius, soleus, and plantaris muscles, epididymal fat pads and liver were excised, weighed, rinsed in saline, clamped and immediately frozen in liquid nitrogen, then wrapped in foil and then stored at −80 °C until further analysis. All analysis was conducted in the Nutrition and Metabolism laboratory at the University of Arkansas.

### 2.2. Plasma Analysis

Plasma glucose was determined using a commercially available kit (Cayman Chemicals, Ann Arbor, MI, USA; catalog #10009582). Insulin was determined using a commercially available ELISA (Alpco Immunoassays; catalog #80-INSRT-E01). Free fatty acids were determined using a commercially available Free Fatty Acid Fluorometric Assay Kit (Cayman Chemical, Ann Arbor, MI, USA; catalog #700310). Plasma amino acids were analyzed using a commercially available kit (EZ Faast, Phenomenex, Torrance, CA, USA; catalog #KG0-7166) using GC/MS as per manufacturer instructions. Plasma cholesterol (catalog #10007640) and triglycerides (catalog #10010303) were analyzed using commercially available kits (Caymen Chemical, Ann Arbor, MI, USA). Plasma glucagon (catalog #EIAR-GLU-1), IGF-1 (catalog #ELR-IGF1-1) and Leptin (catalog #ELR-LEPTIN-1) was measured using commercially available kits (Ray Biotech, Norcross, GA, USA). Fibroblast growth factor-21 (FGF-21, catalog #MF2100) was also measured using a commercially available kit (R&D Systems, Minneapolis, MN, USA).

### 2.3. Muscle and Liver Histology

Liver fat and muscle fat depositions were measured using an Oil Red O stain (American MasterTech, Lodi, CA, USA; catalog #KTORO), according to manufacturer instructions. Briefly, frozen sections of liver were sliced at 8 µm and frozen sections muscle were sliced at 8 µm using a cryostat (Leica Biosystems, Buffalo Grove, IL, USA). The tissues were fixed to the slides with a five-minute formalin bath and then briefly washed with running tap water. The slides then sat in a bath of isopropanol for two minutes and then were stained in a bath of freshly prepared Oil Red O working solution at 60 °C for ten minutes. After staining there was another bath in 85% isopropanol for one minute followed by nuclei staining with an alum hematoxylin bath for one minute. Finally, the slides were rinsed in tap and distilled water and then mounted in aqueous mountant. The fat droplets were examined and photographed under a microscope. Lipid deposition was quantified using Image J software (NIH, Bethesda, MD, USA).

### 2.4. RNA Isolation and Real-Time PCR

RNA was isolated from muscle tissue using an RNA extraction kit (Norgen, ON, Canada; catalog #37500), in accordance with the manufacturer instructions. RNA was converted to cDNA using an RNA to cDNA conversion kit (Quanta, Gaithersburg, MD, USA; catalog #95047-100). Conversion was done in accordance with manufacturer instruction using a Lightcycler 480 system (Roche, Basal, Switzerland). SYBR green master mix (Quanta, Gaithersburg, MD, USA; catalog #95054-500) was used as the reporter dye for fatty acid synthase (FAS), peroxisome proliferator-activated receptor gamma 1- alpha (PGC1α), peroxisome proliferator-activated receptor gamma (PPARγ), and sirtuin 1 (SIRT1). Then, 18s ribosomal RNA was used as an internal reference gene to normalize data since it remains invariant regardless of treatment [38]. All primers were ordered from IDT (Integrated DNA Technologies, Coralville, IA, USA). Primer sequences can be found in (Supplemental Table S2). All samples and controls were analyzed in duplicate and analyzed using quantitative PCR (ABI 7500 Systems, Grand Island, NY, USA). Relative expressions of target genes were determined using the 2-ΔΔCt method [39].

### 2.5. Western Blot Analysis

Briefly, tissues were powdered using a mortar and pestle kept frozen with liquid nitrogen. For protein sample preparation, samples were homogenized with a Polytron handheld homogenizer, in 7 volumes of homogenization buffer (20 mM HEPES, 2 mM EGTA, 50 mM NaF, 100 mM KCl, 0.2 mM EDTA, 50 mM β-glycerophosphate, 1 mM dithiothreitol, 1 mM benzamidine, 0.5 mM sodium vanadate, and 10 μL/mL protease inhibitor cocktail (all chemical reagents were purchased from Sigma-Aldrich, St. Louis, MO, USA)). The homogenate was immediately centrifuged at 10,000× *g* for 10 min at 4 °C. The supernatant was used to measure total protein content in muscle, liver and adipose tissues using Bradford protein assay (Bio-Rad, Hercules, CA, USA). All antibodies were purchased from Cell Signaling (Beverly, MA, USA). Muscle, liver and adipose tissues were analyzed for p-Akt S473 (catalog #4060S), protein kinase B (Akt), p-4EBP1 T37/46 (catalog #2855S), eukaryotic translation initiation factor 4E binding protein 1 (4EBP1, catalog #9452S), p-P70S6K T389 (catalog # 9432S), p70 ribosomal S6 kinase (P70S6K, catalog #2708S), p-mTOR S2448 (catalog #2971S), mTOR (catalog #2972S), p-AMPK T172 (catalog #2531S), and 5′ AMP-activated protein kinase (AMPK, catalog #2532S) as described in [25,40]. Blots were developed with enhanced chemiluminescence (Bio-Rad, Hercules, CA, USA). Protein expression was analyzed using a ProteinSimple FluorChem M imager (ProteinSimple, Santa Clara, CA, USA), and band density was quantified using Alphaview imaging software (Protein Simple, Santa Clara, CA, USA).

### 2.6. Statistics

Summary statistics were calculated for all data (sample means and sample standard deviations). Repeated measures two-way ANOVA was used to calculate differences in weight gain over time. Two-way ANOVA was used to measure the effect of dietary treatment and body weight (lean versus obese). A Tukey test was used to correct for multiple comparisons. An independent *t*-test was used to analyze differences within dietary treatments (e.g., 20% protein versus 40% protein). Results are reported as means + SEMs. All data analysis was performed using Prism GraphPad Software Version 6.0 (La Jolla, CA, USA). *p* < 0.05 was considered statistically significant.

## 3. Results

### 3.1. Body Weight, Food Intake and Body Composition (Tissue Weights)

**Figure 1:**
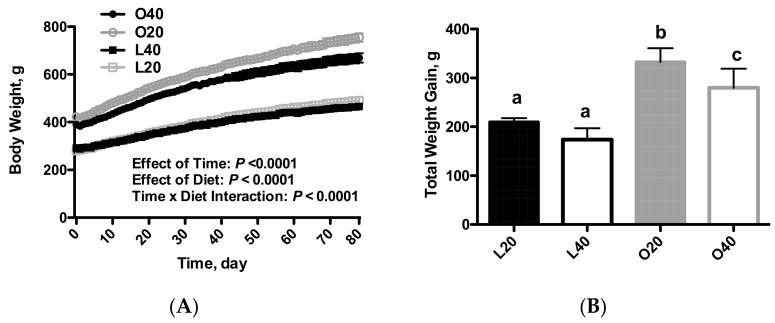

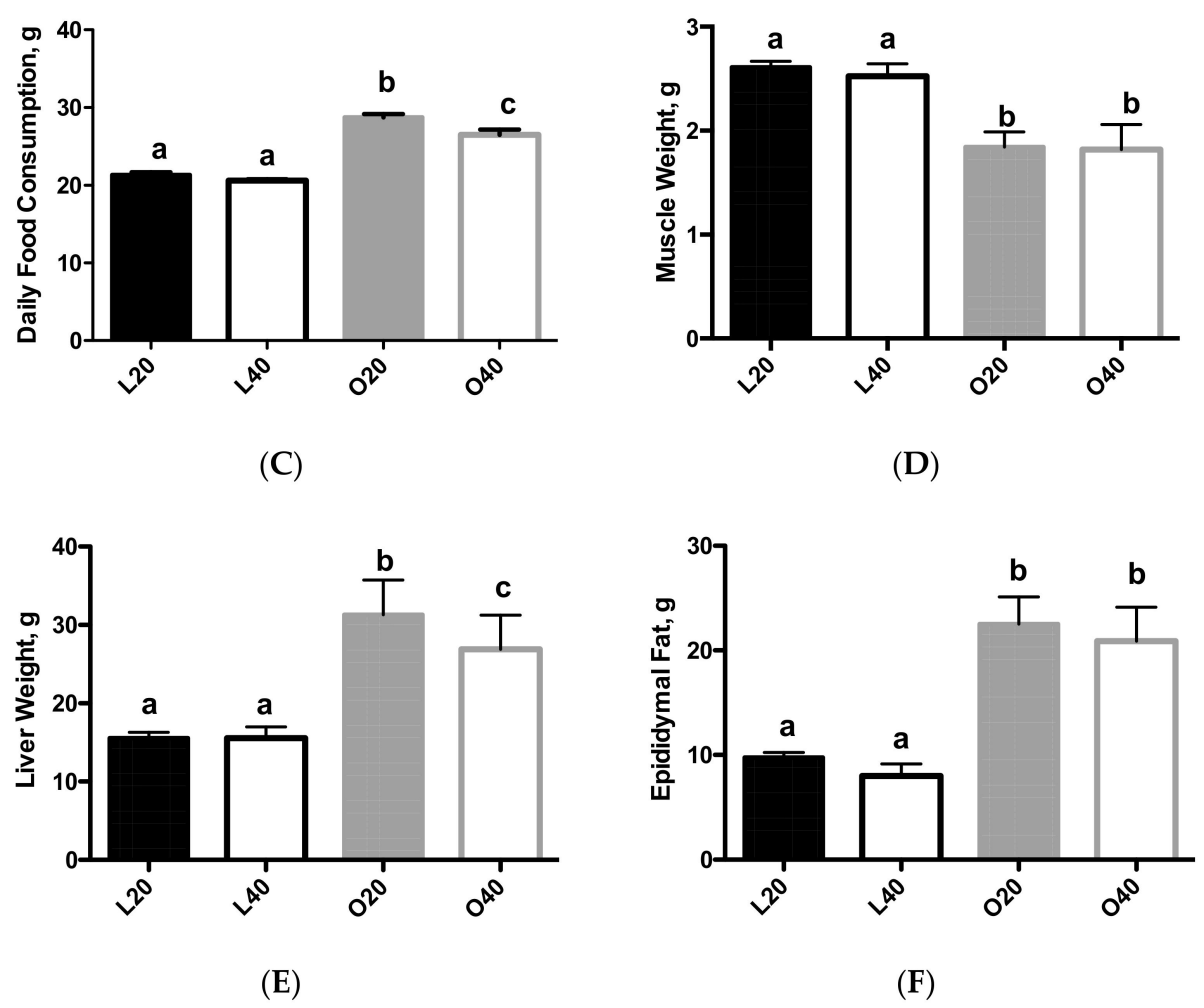
Body weight, food intake and body composition following 12 weeks of consuming either a 20% protein or 40% protein diet in lean control (L) or obese Zucker (O) rats. (**A**) Body weight gain over time; (**B**) Total weight gain over the 12-week diet intervention; (**C**) Average daily food consumption; (**D**) Muscle weight is a combination of gastrocnemius, soleus and plantaris muscles from the right hindlimb; (**E**) Liver weight; (**F**) Epididymal fat pad weight. Values are means + SEM. Data was analyzed using one-way ANOVA. Values without a common letter differ, *p* < 0.05. L20, lean 20% protein; L40, lean 40% protein; O20, obese 20% protein; O40, obese 40% protein.

As expected, the obese Zucker rats had higher body weight gain compared to the lean control rats (Figure 1A). There was an effect of time (*p* < 0.0001), diet (*p* < 0.0001) and time × diet interaction (*p* < 0.0001) for body weight. There was an effect of diet (*p* < 0.0001) and body weight (*p* < 0.0001) on total weight gain. In addition, O40 rats had lower weight gain (*p* < 0.05) over the 12-week dietary intervention period (Figure 1B). This could be due to the reduced food consumption (*p* < 0.05) observed in the O40 versus the O20 (Figure 1C). There was an effect of diet (*p* < 0.01) and body weight (*p* < 0.0001) on food intake. There was an effect of body weight (*p* < 0.001) on muscle and liver weights, with no effect from dietary treatments. Muscle weight (sum of gastrocnemius, plantaris and soleus) was lower in obese versus lean rats (*p* < 0.05; Figure 1D) and epididymal fat weight was higher in obese versus lean rats (*p* < 0.05; Figure 1F), with an effect of body diet (*p* < 0.05) and body weight (*p* < 0.0001). Liver weight was higher in obese versus lean rats (*p* < 0.05; Figure 1E), however O40 had significantly lower liver weights than O20 (*p* < 0.05).

### 3.2. Biomarkers of Obesity and Diabetes

**Figure 2:**
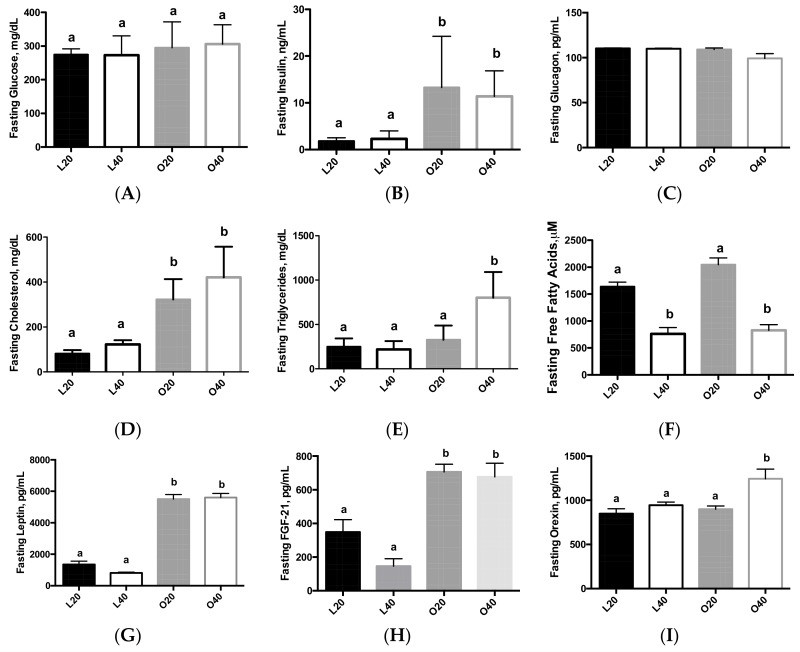
Biomarkers of type 2 diabetes following 12 weeks of consuming either a 20% protein or 40% protein diet in lean control (L) or obese Zucker (O) rats. (**A**) Fasting glucose; (**B**) Fasting insulin; (**C**) Fasting glucagon; (**D**) Fasting cholesterol; (**E**) Fasting triglycerides; (**F**) Fasting free fatty acids; (**G**) Fasting leptin; (**H**) Fasting FGF-21; (**I**) Fasting orexin. Values are means + SEM. Data was analyzed using one-way ANOVA. Values without a common letter differ, *p* < 0.05. FGF-21, fibroblast growth factor-21; L20, lean 20% protein; L40, lean 40% protein; O20, obese 20% protein; O40, obese 40% protein.

There was no difference in fasting blood glucose levels between lean and obese rats (Figure 2A). Fasting insulin levels were higher (*p* < 0.05) in obese versus lean rats, with no effect of diet (Figure 2B). There was no effect of diet or body weight on fasting glucagon levels (Figure 2C). There was an effect of diet (*p* < 0.05) and body weight (*p* < 0.0001) on fasting cholesterol levels (Figure 2D). There was also an effect of diet (*p* < 0.001) and body weight (*p* < 0.0001) on fasting triglyceride levels, with a significant interaction of diet and body weight (*p* < 0.001). Fasting triglyceride levels were higher in O40 rats compared to L20, L40 and O40 (*p* < 0.05; Figure 2D). Both diet (*p* < 0.0001) and body weight (*p* < 0.05) had an effect of fasting free fatty acid (FFA) levels (Figure 2F). Both fasting leptin (Figure 2G) and fasting FGF-21 (Figure 2H) were higher (*p* < 0.05) in the obese, Zucker rats compared to lean rats, with no effect of diet. Fasting orexin (Figure 2I) was significantly higher (*p* < 0.05) in O40 compared to the other three groups. Fasting IGF-1 can be found in (Supplemental Figure S1). Obese Zucker rats had lower (*p* < 0.05) IGF-1 levels than lean rats. Fasting plasma amino acid values are presented in Table 1. There was no effect of body weight on fasting branched chain amino acid levels, however, there was an effect (*p* < 0.05) of dietary protein composition on fasting leucine, isoleucine, and valine concentrations, with rats receiving the 40% protein diet having higher BCAA levels than rats receiving the 20% protein diet. In addition, tryptophan levels were significantly higher (*p* < 0.05) in the lean animals versus the obese animals.

### 3.3. Gene Expression Related to Fat Deposition and Energy Metabolism

**Figure 3:**
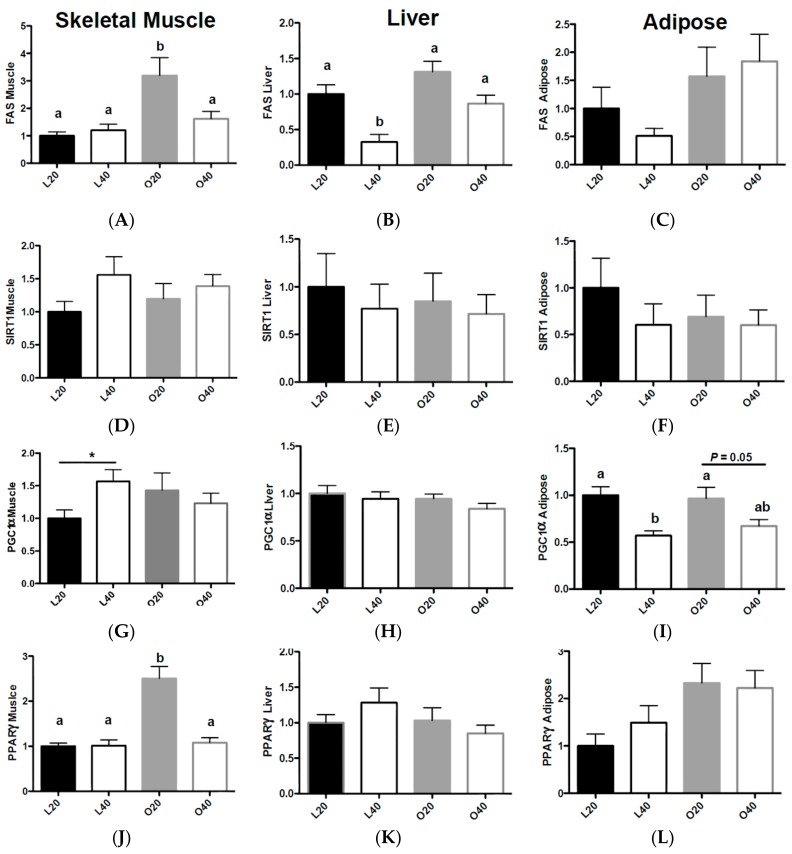
Effects of dietary protein concentration on gene expression in skeletal muscle, liver and epididymal adipose tissue following 12 weeks of consuming either a 20% protein or 40% protein diet in lean control (L) or obese Zucker (O) rats. Relative expressions of target genes were determined using the 2-ΔΔCt method. All genes are expressed relative to 18S, the control gene. (**A**) Fatty acid synthase (FAS)expression in skeletal muscle; (**B**) FAS expression in liver; (**C**) FAS expression in adipose tissue; (**D**) Sirtuin 1 (SIRT1) expression in skeletal muscle; (**E**) SIRT1 expression in liver; (**F**) SIRT1 expression in adipose tissue; (**G**) Peroxisome proliferator-activated receptor-γ coactivator-1α (PGC1α) expression in skeletal muscle; (**H**) PGC1α expression in liver; (**I**) PGC1α expression in adipose tissue; (**J**) Peroxisome proliferator-activated receptor-γ (PPARγ) expression in skeletal muscle; (**K**) PPARγ expression in liver; (**L**) PPARγ expression in adipose tissue. Values are means + SEM. Data was analyzed using one-way ANOVA. Values without a common letter differ, *p* < 0.05. Values with an * were analyzed via *t*-test and are significantly different within a group (20% versus 40% protein), *p* < 0.05. L20, lean 20% protein; L40, lean 40% protein; O20, obese 20% protein; O40, obese 40% protein.

Gene expression data is depicted in Figure 3. FAS expression in skeletal muscle was higher (*p* < 0.05) in O20 versus L20, L40 and O40 muscle (Figure 3A). There was an effect of body weight and diet–body weight interaction on skeletal muscle fatty acid synthase (FAS) (*p* < 0.05). There was an effect of diet and body weight (*p* < 0.01) on liver FAS expression. Liver L40 had lower (*p* < 0.05) FAS expression (Figure 3B) compared to L20, O20 and O40. There was no effect of diet on adipose tissue FAS expression, however there was a significant (*p* < 0.05) effect of body weight (Figure 3C). There was no difference on SIRT1 expression in skeletal muscle, liver or adipose tissue (Figure 3D–F). There was an increase (*p* < 0.05) in PGC1α expression (Figure 3G) in skeletal muscle in L40 versus L20 animals, no difference in PGC1α between dietary treatments in liver (Figure 3E), and a significant effect (*p* < 0.001) of diet on PGC1α expression in adipose tissue. L40 and O40 animals had significantly lower PGC1α expression (*p* < 0.05) compared to L20 and O20 in adipose tissue (Figure 3I). There was a significant effect of diet (*p* < 0.001), body weight (*p* < 0.001) and diet × body weight interaction (*p* < 0.001) for skeletal muscle PPARγ (Figure 3J) expression. PPARγ was significantly higher (*p* < 0.05) in skeletal muscle of O20 versus L20, L40 and O40 animals, with no effect of diet or body weight in liver PPARγ expression (Figure 3K). There was an effect of body weight (*p* < 0.05) on PPARγ expression in adipose tissue (Figure 3L).

### 3.4. Tissue Neutral Lipid Deposition

**Figure 4:**
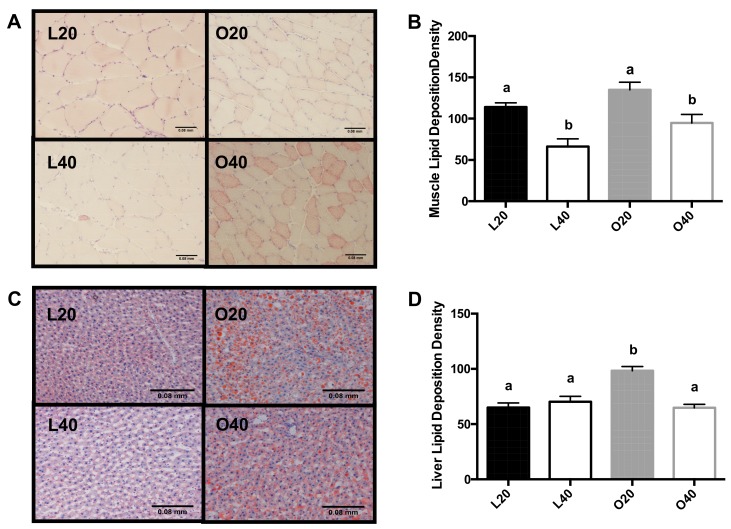
Representative skeletal muscle (gastrocnemius; 8 µm thickness and 200× magnification; 4.17 pixels/um) and liver sections (12 µm thickness and 200× magnification; 4.17 pixels/um) were obtained from lean control (L) or obese Zucker (O) rats following 12 weeks of consuming either a 20% protein or 40% protein diet and stained for fat deposition using Oil Red O staining. (**A**) Skeletal muscle lipid deposition, scale indicates 0.08 mm; (**B**) Quantification of lipid deposition in skeletal muscle, scale indicates 0.08 mm; (**C**) Liver lipid deposition; (**D**) Quantification of lipid deposition in liver. Obese rats had higher fat deposition in muscle and liver compared to lean rats. Rats receiving the 40% protein diet tended to have less fat deposition than those fed the 20% protein diet. L20, lean 20% protein; L40, lean 40% protein; O20, obese 20% protein; O40, obese 40% protein.

Skeletal muscle and liver lipid deposition are depicted in Figure 4A,B, respectively. L40 and O40 animals had lower skeletal muscle and liver lipid deposition compared to L20 and O20 animals, respectively.

### 3.5. Metabolic Signaling

**Figure 5:**
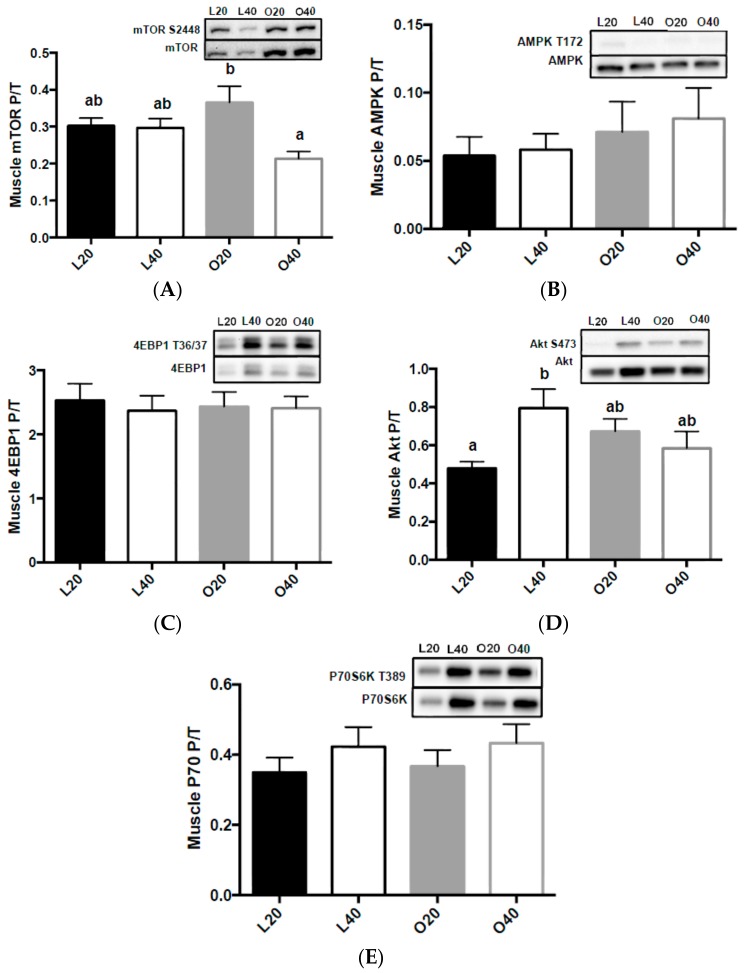
Changes in mTORC1 signaling in gastrocnemius muscle following 12-weeks of consuming either a 20% protein (*n* = 8 per group) or 40% protein diet (*n* = 10 per group) in lean control (L) or obese Zucker (O) rats. Representative blots are shown, all bands are from the same blot. (**A**) Mammalian target of rapamycin complex 1 (mTORC1) phosphorylation (S2448) over total protein; (**B**) 5′ AMP-activated protein kinase (AMPK) phosphorylation (T172) over total protein; (**C**) Eukaryotic translation initiation factor 4E binding protein 1 (4EBP1) phosphorylation (S65) over total protein; (**D**) Protein kinase B (Akt) phosphorylation (S473) over total protein; (**E**) Ribosomal protein S6 kinase 1 (P70) phosphorylation (T389) over total protein. Values are means + SEM. Data was analyzed using one-way ANOVA. Values without a common letter differ, *p* < 0.05. L20, lean 20% protein; L40, lean 40% protein; O20, obese 20% protein; O40, obese 40% protein; P/T, phosphorylated/total protein.

There was an effect of diet (*p* < 0.05) and a significant interaction of diet x body weight (*p* < 0.05) on skeletal muscle mTORC1 phosphorylation. Skeletal muscle mTORC1 phosphorylation (Figure 5A) was higher (*p* < 0.05) in muscle of O20 animals compared to L20, L40 and O40; there was no change in AMPK (Figure 5B), 4EBP1 (Figure 5C), or p70S6K (Figure 5E) phosphorylation between groups. However, there was a significant (*p* < 0.05) interaction between diet and body weight on skeletal muscle Akt phosphorylation. L40 had significantly higher Akt phosphorylation than L20 (Figure 5D).

**Figure 6:**
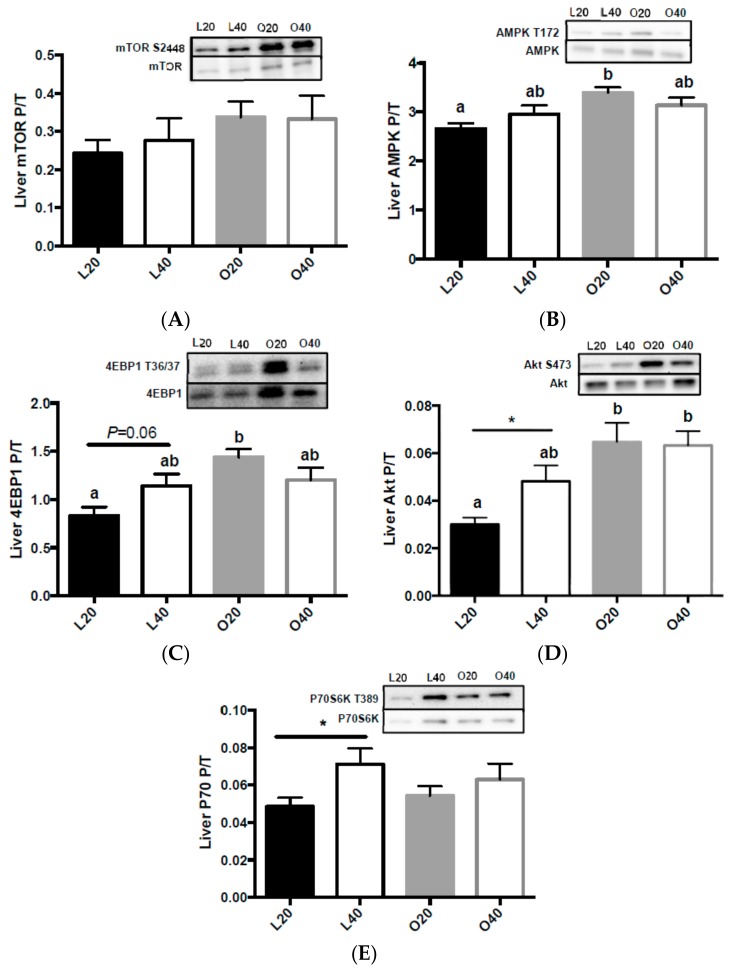
Changes in mammalian target of rapamycin complex 1 (mTORC1) signaling in liver following 12 weeks of consuming either a 20% protein (*n* = 8 per group) or 40% protein diet (*n* = 10 per group) in lean control (L) or obese Zucker (O) rats. Representative blots are shown, all bands are from the same blot. (**A**) Mammalian target of rapamycin complex 1 (mTORC1) phosphorylation (S2448) over total protein; (**B**) 5′ AMP-activated protein kinase (AMPK) phosphorylation (T172) over total protein; (**C**) Eukaryotic translation initiation factor 4E binding protein 1 (4EBP1) phosphorylation (S65) over total protein; (**D**) Protein kinase B (Akt) phosphorylation (S473) over total protein; (**E**) Ribosomal protein S6 kinase 1 (P70) phosphorylation (T389) over total protein. Values are means + SEM. Data was analyzed using one-way ANOVA. Values without a common letter differ, *p* < 0.05. Values with an * were analyzed via *t*-test and are significantly different within a group (20% versus 40% protein), *p* < 0.05. L20, lean 20% protein; L40, lean 40% protein; O20, obese 20% protein; O40, obese 40% protein; P/T, phosphorylated/total protein.

In liver, there was no difference in mTORC1 phosphorylation between groups (Figure 6A). There was an effect (*p* < 0.01) of body weight on liver AMPK phosphorylation. O20 had higher (*p* < 0.05) liver AMPK phosphorylation compared to L20 (Figure 6B). There was a significant effect of body weight (*p* < 0.01) on 4EBP1 phosphorylation and a significant interaction of diet and body weight (*p* < 0.05). L40 had increased 4EBP1 (Figure 6C), Akt (Figure 6D) and p70S6K (Figure 6E) phosphorylation compared to L20. There was an effect (*p* < 001) of body weight on liver Akt phosphorylation (Figure 6D) and no effect of either diet or body weight on p70S6K phosphorylation.

**Figure 7:**
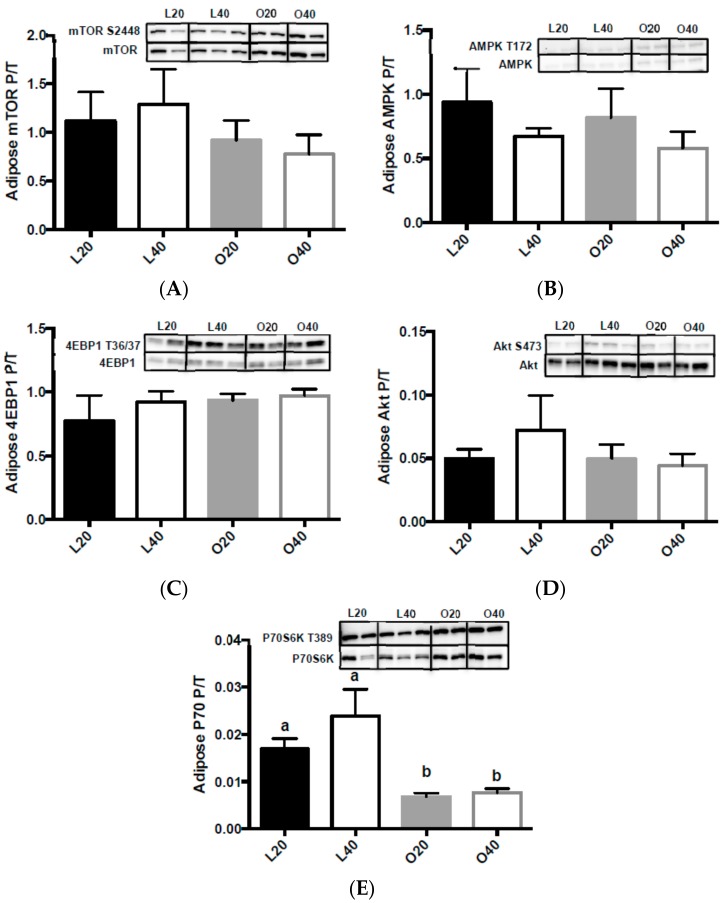
Changes in mammalian target of rapamycin complex 1 (mTORC1) signaling in epididymal adipose tissue following 12-weeks of consuming either a 20% protein (*n* = 8 per group) or 40% protein diet (*n* = 10 per group) in lean control (L) or obese Zucker (O) rats. Representative blots are shown, all bands are from the same blot. (**A**) Mammalian target of rapamycin complex 1 (mTORC1) phosphorylation (S2448) over total protein; (**B**) 5′ AMP-activated protein kinase (AMPK) phosphorylation (T172) over total protein; (**C**) Eukaryotic translation initiation factor 4E binding protein 1 (4EBP1) phosphorylation (S65) over total protein; (**D**) Protein kinase B (Akt) phosphorylation (S473) over total protein; (**E**) Ribosomal protein S6 kinase 1 (P70) phosphorylation (T389) over total protein. Values are means + SEM. Data was analyzed using one-way ANOVA. Values without a common letter differ, *p* < 0.05. L20, lean 20% protein; L40, lean 40% protein; O20, obese 20% protein; O40, obese 40% protein; P/T, phosphorylated/total protein. Figure without boxes separating treatment groups can be found in Supplementary Data Figure S2.

There was no significant change in adipose mTOR (Figure 7A), AMPK (Figure 7B), 4EBP1 (Figure 7C), or Akt (Figure 7D) phosphorylation. There was an effect (*p* < 0.001) of body weight on adipose p70S6K phosphorylation in adipose tissue. Adipose p70S6K phosphorylation was significantly higher (*p* < 0.05) in lean versus obese animals (Figure 7E).

## 4. Discussion

To our knowledge, this is one of the first studies to demonstrate that a higher protein diet delays the onset of obesity in male, Zucker rats. These results support the hypothesis that high-protein diets decrease weight gain, improve muscle function, and improve select biomarkers of T2DM compared to a moderate-protein diet. Obese rats receiving the high-protein diet had reduced weight gain over the twelve-week feeding period, as well as a significantly reduced amount of daily food consumption. The obese rats on the high-protein diet also exhibited lower liver weights and less fat deposition in the liver, compared to those receiving the moderate-protein diet. In addition, obese rats on the high-protein diet had decreased PPARγ expression and decreased mTOR activation compared to the obese rats on the moderate-protein diet. There was also a significant decrease in plasma FFA, increase in plasma BCAA, and decrease in plasma tryptophan compared to the rats consuming the moderate protein diet.

The decrease in weight gain was an expected result of the higher protein diet as many studies have shown that increasing protein in the diet reduces weight gain compared to diets lower in protein [4,7,41]. The decreased weight gain could be a direct result of decreased food intake also observed or due to the mutation in the leptin receptor gene that is found in Zucker rats. Although the differences in food intake observed between the 20% versus 40% protein diet could be due to taste (the 40% protein diet contained 200 g less sucrose per kg), several studies have shown that increasing protein in the diet reduces hunger and increases feelings of fullness, which can result in decreased food intake [27,42,43,44,45]. The direct cause of protein-induced satiation is still unclear, but it may be a cumulative effect of different factors. Studies have shown that rats become satiated when they ingest increased amounts of limiting or non-limiting amino acids [45,46]. The mechanism for this is not well understood; however, studies have shown that increased amino acids stimulate vagal feedback to the nucleus tractus solitarius and the hypothalamus, which are both areas involved with hunger and satiation [27]. The obese rats in this study had significantly lower levels of plasma tryptophan. Tryptophan is a precursor for the neurotransmitter serotonin, which modulates appetite [27,47], which could explain the increase in food intake in obese versus lean rats. Diets high in protein also tend to be high in the BCAA leucine. Centrally-administered leucine decreases food intake and body weight in mice, via an mTORC1-mediated pathway [48]. In addition, rodents with diet-induced obesity receiving leucine supplementation, have decreased weight gain due to decreased food intake and increased energy expenditure (reviewed by [23,49]). Another option is that the decreased weight gain could be due to increased thermic effect of feeding (TEF), however we were not able to measure TEF in this study. It is also important to note that obese Zucker rats have disruption of the leptin axis, which could also be responsible for altering food intake and hunger control [50].

We observed significantly lower liver weights in obese animals on the high protein diet (O40). The lower liver weights could be attributed to an increased hepatic regulation of blood glucose. Increased protein in the diet has been shown to be consistent with an increase in hepatic glucose production [9,42]. Many parts of the body, including skeletal muscle, use FFAs to produce ATP in the absence or inability to use glucose. In the case of obese Zucker rats, it has been shown that these animals exhibit increased hepatic lipogenesis in compensation for the lack of energy being received from glucose [51]. With the decreased amount of fasting plasma FFAs and the visible reduction of fat in the liver, our results show that a high-protein diet could be due to decreased hepatic lipogenesis, as indicated by the decrease in liver FAS expression, the rate-limiting enzyme in de novo fatty acid synthesis, in these animals. This increase in the use of the liver for glucose storage and production, coupled with a decrease in hepatic lipogenesis, could be a possible reason for the observed decrease in weight and fat in the liver. Previously it has been shown that high-protein diets have the ability to reduce fat mass and improve glucose tolerance in Western-type diet-induced obese rats [52], however diets using diet-induced obesity to study the effects of high-protein diets are limited.

Fasting FFAs were significantly lower in animals on the high-protein diet. This could be a sign of increased insulin sensitivity because insulin resistance is associated with high levels of circulating fatty acids, which can inhibit the insulin-signaling pathway [53,54], which was observed in the lean moderate-protein versus lean high-protein-fed animals. The L40 rats had significantly higher muscle and liver Akt phosphorylation compared to the L20 animals. The reason for the decrease in FFAs is unclear, however it may be related to increased oxidation of fat during fasting. The lower levels of FFA during fasting could be a sign of improved metabolic flexibility, which is defined as an organism’s ability to switch from glucose oxidation under postprandial conditions to fat oxidation during fasting conditions [54]. There was no difference in fasting plasma glucose between lean or obese animals or between dietary treatments. These findings are supported by studies in humans [55,56], which have demonstrated that long-term high-protein diets have little effect on fasting plasma glucose levels, as well as in animal studies [57]. Although we did not collect postprandial data in this study, Bernard et al. [58] have shown that an amino acid mixture (high in the BCAA leucine) can improve glucose tolerance and decrease insulin resistance in obese, Zucker rats in an acute setting. This suggests that diets high in protein (and BCAA) may have the potential to improve postprandial glucose and insulin response under obese, diabetic conditions.

Animals receiving the high protein diet had significantly higher fasting plasma BCAA levels. This could be a positive sign as elevated circulating levels of BCAA have been shown to reverse the effects of oxidative stress which contributes to impaired insulin secretion and the development of T2DM [59]. The benefits of BCAAs are not universally accepted, however, and many studies have shown negative correlations for elevated circulating BCAA and overall health (reviewed by [60]). Elevated BCAAs have been associated with increased metabolic diseases, including T2DM and obesity [60,61,62,63]. However, the association between elevated BCAA and poor metabolic health may only occur when coupled with a high-fat diet [42]. She et al. [63] suggest that the increased levels of BCAA observed in Zucker rats could be a result of increased food intake; however, our results contradict this and suggest that it is increased BCAA in the diet that is associated with higher levels of circulating BCAA and not total food intake.

Chronic overactivation of mTORC1, which can occur with chronic nutrient surplus, is linked to metabolic diseases such as obesity and type 2 diabetes [64,65]. We observed increased muscle mTORC1 activation under fasting conditions in obese animals fed the moderate-protein diet; this was ameliorated in muscle from obese animals fed a high-protein diet. In muscle [32,66], liver [25,40] and adipose tissue [67] mTORC1 has been shown to regulate the protein synthesis by phosphorylating the eukaryotic initiation factor binding protein 4E, which signals translation initiation, as well as activating p70 ribosomal S6 kinase (p70S6K), a key mediator of the protein synthesis cascade [35]. In this study, obese animals had significantly lower hindlimb muscle mass. It is well established that the obese Zucker rat has relatively smaller skeletal muscle mass compared to the lean Zucker rat skeletal muscle [51,68,69]. This cannot be explained by changes in muscle mTORC1, 4EBP1 or p70S6K phosphorylation. Activation of mTORC1 in muscle of obese and high-fat-fed rodents results in p70S6K-mediated feedback inhibition of insulin signaling, which can reduce glucose update by muscle and contribute to systemic insulin resistance [35]. This impaired insulin signaling could contribute to the muscle mass loss observed in obesity and insulin resistance, as well as the decreased muscle mass observed in obese animals in this study, by promoting protein catabolism [35,70], although more analysis is needed to confirm this mechanism in the present study.

Data suggest that mTORC1 can also mediate adipogenesis via PPAR-γ [36,37], a master regulator of adipogenesis and lipid metabolism [37]. mTORC1 has also been associated with PPARγ-induced stimulation of adipose tissue lipid uptake and fat accretion [37]. In this study, mTORC1 phosphorylation was increased in O20 rats compared to L20, L40 and O40 rats. An increase in PPARγ was also observed in O20 rats, suggesting that mTORC1 may be regulating skeletal muscle adipose deposition via PPARγ. Finally, PGC1α (PPARγ coactivator) is recognized as a key regulator of tissue metabolism by upregulating mitochondrial biogenesis, hepatic gluconeogenesis and muscle glucose transport [71]. Benton et al. [71] found that when PGC1α expression is upregulated when triacyclglycerol synthesis rates are reduced in skeletal muscle. In this study, we found PGC1α expression to be upregulated in L40 compared to L20 animals. In addition, we found decreased neutral lipid stain in L40 versus L20 animals, which is indicative of decreased intramyocellular lipid deposition, however this was not quantified.

## 5. Limitations

Although the data support the benefits of higher protein diets, there are limitations to this study. First, we did not use female rats in this study. Data suggest that diets higher in protein may influence fat loss to a greater extent in males versus females [72], which could affect the conclusions drawn from this study. In addition, the moderate protein (20% energy from protein) contains a higher protein intake than the standard AIN-93M diet (14% energy from protein) [73], which means both diets used in this study could be considered higher protein when compared to the animals’ needs. Expression of fatty acid synthase was the only enzyme measured in this study; this is a limitation because it is only one marker of lipogenesis. Inclusion of expression of additional enzymes such as acetyl-CoA carboxylase (ACC) and 11β-hydroxysteroid dehydrogenase type 1 (11βHSD1) could also help with further defining and identifying mechanisms of action. Another limitation is that we did not measure body temperature or energy expenditure, which could provide insight into the mechanisms of action related to weight gain in 20% protein-fed versus 40% protein-fed animals. Finally, plasma measurements were only conducted after an overnight fast therefore we do not know the postprandial meal response, which is important for understanding glucose and fat metabolism [9].

## 6. Conclusions

We conclude that high-protein diets have the potential to reduce weight gain and alter metabolism in obese Zucker rats, possibly through regulation of mTORC1-dependent pathway in skeletal muscle. However, further research is needed to determine the specific mechanisms of action.

## Figures and Tables

**Table 1:** Fasting plasma amino acid composition ^1^.

	L20	L40	O20	O40	Effect of Diet (20% v 40% Protein)	Effect of Body Weight (Lean v Obese)	Interaction Diet *X* Body Weight
Alanine	399 ± 26	327 ± 25	380 ± 39	381 ± 25	*ns*	*ns*	*ns*
Glycine	135 ± 8 ^a^	95 ± 4 ^b^	98 ± 5 ^b^	64 ± 3 ^c^	<0.0001	<0.0001	*ns*
Valine	167 ± 9 ^a^	194 ± 10 ^a,b^	161 ± 10 ^a^	232 ± 14 ^b^	<0.001	*ns*	*ns*
Leucine	112 ± 6 ^a,b^	135 ± 5 ^a,c^	108 ± 7 ^b^	151 ± 9 ^c^	<0.0001	*ns*	*ns*
Isoleucine	60 ± 3 ^a^	69 ± 2 ^a,b^	59 ± 4 ^a^	81 ± 5 ^b^	<0.001	*ns*	*ns*
Threonine	277 ± 11 ^a,b^	230 ± 8 ^a,c^	309 ± 34 ^b^	187 ± 9 ^c^	<0.0001	*ns*	<0.05
Serine	163 ± 6 ^a,b^	151 ± 5 ^a,b^	168 ± 11 ^a^	142 ± 5 ^b^	<0.01	*ns*	*ns*
Proline	259 ± 11 ^a,^	189 ± 11 ^b^	141 ± 13 ^c^	144 ± 10 ^c^	<0.05	<0.0001	<0.01
Asparagine	35 ± 2 ^a^	30 ± 1 ^b^	28 ± 2 ^b^	25 ± 1 ^b^	<0.01	<0.001	*ns*
Aspartate	8.6 ± 0.6	8.2 ± 0.4	7.9 ± 0.6	7.9 ± 0.5	*ns*	*ns*	*ns*
Methionine	36 ± 2 ^a^	25 ± 2 ^b^	25 ± 3 ^b^	23 ± 1 ^b^	<0.01	<0.01	*<0.05*
Glutamate	132 ± 8 ^a^	107 ± 4 ^a,b^	115 ± 7 ^a,b^	93 ± 7 ^b^	<001	<0.05	*ns*
Phenylalanine	52 ± 3	54 ± 3	56 ± 3	57 ± 3	*ns*	*ns*	*ns*
Glutamine	493 ± 36 ^a^	778 ± 73 ^b,d^	573 ± 38 ^b,c^	825 ± 36 ^d^	<0.0001	*ns*	*ns*
Lysine	521 ± 40 ^a,b,c^	609 ± 17 ^a,c^	476 ± 33 ^b^	622 ± 31 ^c^	<0.001	*ns*	*ns*
Histidine	44 ± 4	43 ± 3	49 ± 2	45 ± 2	*ns*	*ns*	*ns*
Tyrosine	68 ± 5 ^a,b^	53 ± 4 ^a^	74 ± 7 ^b^	52 ± 2 ^a^	<0.001	*ns*	*ns*
Tryptophan	95 ± 6 ^a^	83 ± 4 ^a^	58 ± 5 ^b^	52 ± 4 ^b^	<0.05	<0.0001	*ns*

^1^ Values are means + SEM. Within a row, values without a common letter differ, *p* < 0.05; ns, not significant (*p* > 0.05). L20, lean 20% protein; L40, lean 40% protein; O20, obese 20% protein; O40, obese 40% protein. 2 *p*-value determined by two-way ANOVA.

## Supplementary Materials

The following are available online at www.mdpi.com/2072-6643/9/6/587/s1, Figure S1: Fasting Insulin-like Growth Factor 1, Figure S2: Without boxes around Western blots, Table S1: Experimental Diet Composition, Table S2: Primer Sequences1.

## Abbreviations

4E-BP1	eukaryotic initiation 4E factor binding protein 1
ADMR	acceptable macronutrient distribution range
Akt	protein kinase B
AMPK	AMP-activated protein kinase
FAS	fatty acid synthase
FGF-21	fibroblast growth factor-21
IGF-1	insulin-like growth factor-1
L20	lean animals on 20% protein diet
L40	lean animals on 40% protein diet
mTORC1	mechanistic target of rapamycin complex 1
O20	obese animals on 20% protein diet
O40	obese animals on 40% protein diet
p70S6K	ribosomal protein S6 kinase
PGC1α	peroxisome proliferator-activated receptor gamma coactivator 1-alpha
PPARγ	peroxisome proliferator-activated receptor gamma
SIRT1	sirtuin 1
