# Supplementary material for: A High-Protein Diet Reduces Weight Gain, Decreases Food Intake, Decreases Liver Fat Deposition, and Improves Markers of Muscle Metabolism in Obese Zucker Rats
Source: Nutrients. 2017 Jun 8;9(6):587. doi: 10.3390/nu9060587 (PMC5490566; doi:10.3390/nu9060587)
Supplement: Supplementary file 1 [file nutrients-09-00587-s001.zip › nutrients-179781-supplementary.pdf]

Online Supplementary Material

Supplemental Table S1. Experimental Diet Composition[1]

Ingredient (g/kg)	20% Protein[2]	40% Protein[3]
Casein	230	460
DL-Methionine	3	-
Sucrose	431.7	231.82
Corn Starch	200	200
Corn Oil	52.3	50
Cellulose	37.86	15
Vitamin Mix	10	10
Ethoxyquin, antioxidant	0.01	0.01
Mineral Mix	13.37	13.37
Calcium Phosphate, dibasic	16.66	9.6
Calcium Carbonate	5.1	10.2
kcal/g	3.8	3.8

[1]Experimental diets purchased pre-pelleted and pre-formulated.

[2]Teklad Diets (TD.91352), Envigo RMS, Indianapolis, IN

[3]Teklad Diets (TD.90018), Envigo RMS, Indianapolis, IN.

Supplemental Table S2. Primer Sequences[1]

rFAS Forward	5' - CAGCTGTCAGTGTAAAGAAACATGTC - 3'
rFAS Reverse	5' - AGCTCACGTGCAGTTTAATTGTG - 3'
rSIRT1 Forward	5' - TGGTAGAGCCTCCATAGATCTTCA - 3'
rSIRT1 Reverse	5' - TAGGGTCAGAAAGGCTTTGCA - 3'
rPGC-1α Forward	5' - TGAAGAGCGCCGTGTGATT -3'
rPGC-1α Reverse	5' - TTCTGTCCGCGTTGTGTCA -3'
rPPARγ1 Forward	5' - CCCACCAACTTCGGAATCAG - 3'
rPPARγ 1 Reverse	5' - GGAATGGGAGTGGTCATCCA - 3'
r18S Forward	5' - TTCGTATTGCGCCGCTAGA - 3'
r18S Reverse	5' - CTTTCGCTCTGGTCCGTCTT - 3'

[1]National Center for Biotechnology Information (NCBI) Entrez Gene

Supplemental Figure S1

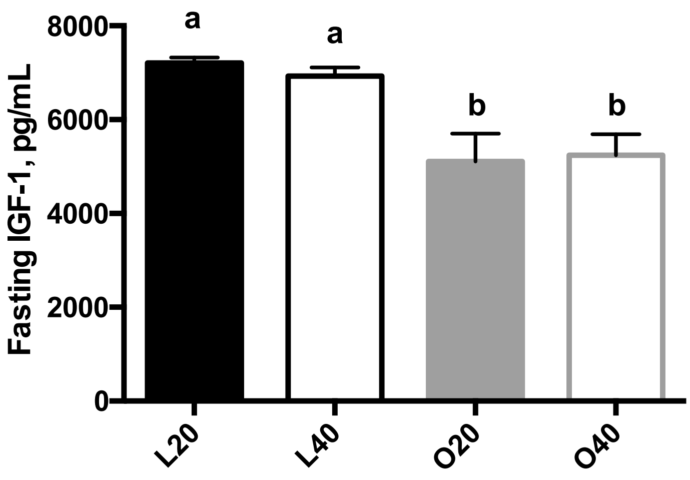

Supplemental Figure S2

Figure 7 (without boxes around Western blots)

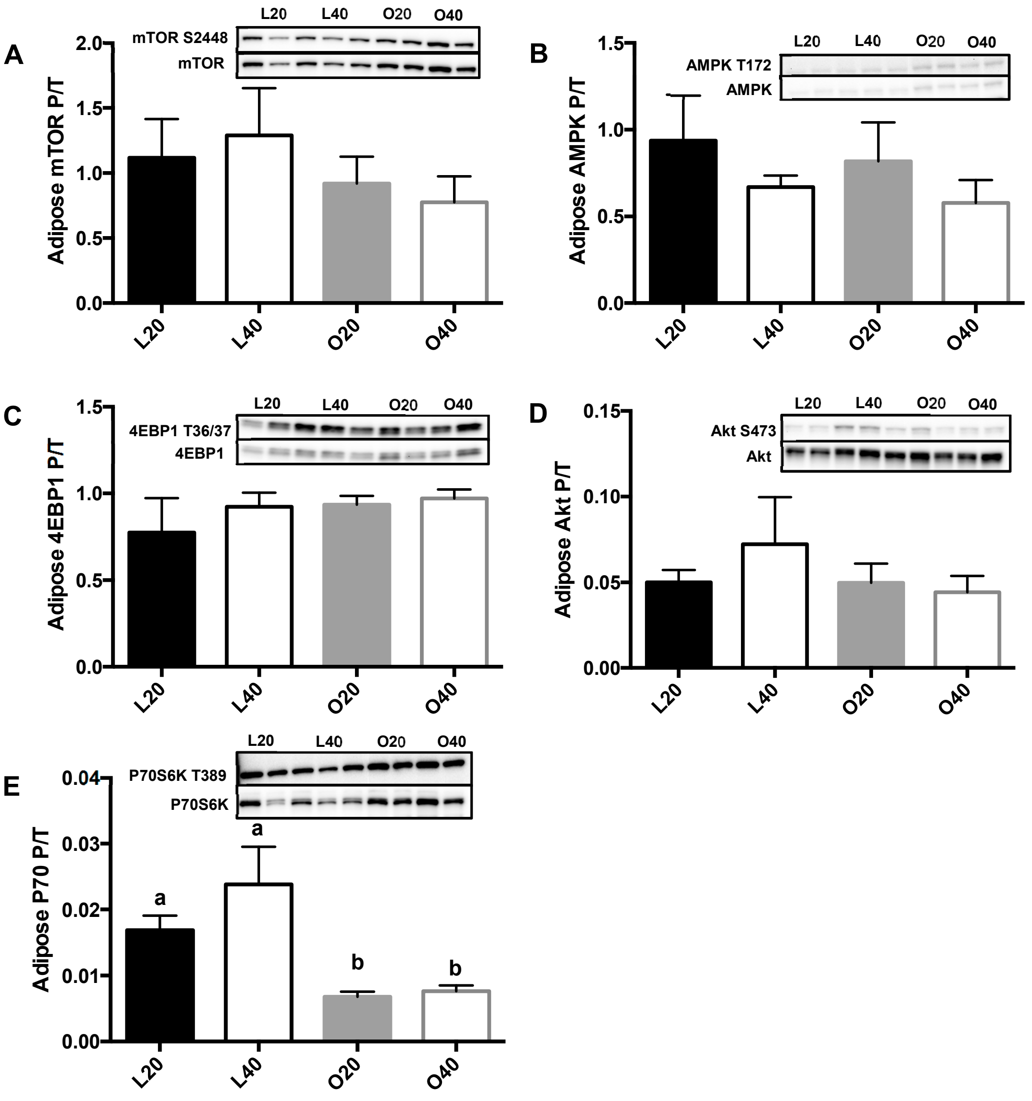

Figure 7. Changes in mTORC1 signaling in epididymal adipose tissue following 12-weeks of consuming either a 20% protein (n=8 per group) or 40% protein diet (n=10 per group) in Lean control (L) or Obese Zucker (O) rats. Representative blots are shown, all bands are from the same blot. A. Mammalian target of rapamycin complex 1 (mTORC1) phosphorylation (S2448) over total protein. B. 5' AMP-activated protein kinase (AMPK) phosphorylation (T172) over total protein. C. Eukaryotic translation initiation factor 4E binding protein 1 (4EBP1) phosphorylation (S65) over total protein. D. Protein kinase B (Akt) phosphorylation (S473) over total protein. E. Ribosomal protein S6 kinase 1 (P70) phosphorylation (T389) over total protein. Values are means ± SEM. Data was analyzed using one-way ANOVA. Values without a common letter differ, $P < 0.05$. Values with an * were analyzed via t-test and are significantly different within a group (20% versus 40% protein), $P < 0.05$. L20, Lean 20% protein; L40, Lean 40% protein; O20, Obese 20% protein; O40, Obese 40% protein; P/T, phosphorylated/total protein. Figure without boxes separating treatment groups can be found in Supplementary Data.
